# Role of Soluble Innate Effector Molecules in Pulmonary Defense against Fungal Pathogens

**DOI:** 10.3389/fmicb.2017.02098

**Published:** 2017-10-31

**Authors:** Soledad R. Ordonez, Edwin J. A. Veldhuizen, Martin van Eijk, Henk P. Haagsman

**Affiliations:** Division of Molecular Host Defence, Department of Infectious Diseases and Immunology, Faculty of Veterinary Medicine, Utrecht University, Utrecht, Netherlands

**Keywords:** antimicrobial peptides, pulmonary collectin, lung defense, innate immunity, antifungal defenses

## Abstract

Fungal infections of the lung are life-threatening but rarely occur in healthy, immunocompetent individuals, indicating efficient clearance by pulmonary defense mechanisms. Upon inhalation, fungi will first encounter the airway surface liquid which contains several soluble effector molecules that form the first barrier of defense against fungal infections. These include host defense peptides, like LL-37 and defensins that can neutralize fungi by direct killing of the pathogen, and collectins, such as surfactant protein A and D, that can aggregate fungi and stimulate phagocytosis. In addition, these molecules have immunomodulatory activities which can aid in fungal clearance from the lung. However, existing observations are based on *in vitro* studies which do not reflect the complexity of the lung and its airway surface liquid. Ionic strength, pH, and the presence of mucus can have strong detrimental effects on antifungal activity, while the potential synergistic interplay between soluble effector molecules is largely unknown. In this review, we describe the current knowledge on soluble effector molecules that contribute to antifungal activity, the importance of environmental factors and discuss the future directions required to understand the innate antifungal defense in the lung.

## Introduction

At the lung lining, several innate defenses are present that contribute to prevention of fungal infection. These include mucociliary clearance of fungi, production of soluble effector molecules with antifungal activity and/or immune stimulating activity, and roaming phagocytic cells that can neutralize incoming pathogens. However, understanding how these molecular defenses act in concert to prevent infection in a healthy lung has proven to be difficult. Usually, studies are performed on the antifungal activity of single components (either defense molecules or cells) which does not necessarily reflect their activity in a complex mixture due to either antagonistic or synergistic activities. Moreover, effector molecules may have multiple functions that may further complicate their individual contribution in lung host defense. Adding to this challenge is the difficulty of mimicking the complex conditions of the lung environment *in vitro*. At the lung lining, several factors such as pH, ionic strength, and mucus concentration will also affect the activity of many innate immune components.

The study of immune defenses is most often approached using infection models. Even though these models yield useful information, they do not fully apply to a healthy lung, where the fungal intruders need to be controlled without causing an excessive immune response. In this review, we will first summarize the current knowledge of the antifungal function of soluble effector molecules. Then we will discuss how the interaction of these molecules with epithelial cells and phagocytes modulates protection against fungal pathogens. Finally, the possibility of concerted activities between these molecules and their interactions with the lung environment will be addressed.

### Soluble Effector Molecules

Soluble effector molecules are known to exert their antifungal function in three ways: (1) direct killing, (2) opsonization, and (3) immunomodulation. Direct killing is classically known as the main function of host defense peptides (HDPs), but other soluble molecules such as enzymes can also directly target fungal cells. Opsonization on the other hand is an important function of collagen-containing C-type lectins (collectins). HDPs and collectins can also modulate the immune response of epithelial and phagocytic cells, with respect to, for example, cytokine production and activation status of immune cells. These immunomodulatory properties are significantly contributing to an effective overall defense strategy of the host. The three different modes of antifungal activity expressed by soluble effectors will be reviewed in the following sections.

#### Direct Killing

Several constitutively secreted proteins and peptides line the lung inner surface and many of them have shown to exert direct fungicidal activity *in vitro*. One of the most abundant proteins with fungicidal activity is lysozyme, a small protein (approximately 15 kDa) produced by epithelial cells and alveolar macrophages in the human lung [10 μg/ml in bronchoalveolar lavage fluid (BALF)] (Dubin et al., 2004). Lysozyme is one of the first characterized antimicrobial proteins, discovered by Alexander Fleming in 1922. Its antimicrobial activity involves two distinct mechanisms (Düring et al., 1999). First, the enzymatic cleavage of the N-glycosidic bonds linking polysaccharides and proteins in the bacterial cell wall and chitodextrins in fungal cell walls. Second, the permeabilization of the plasma membrane, due to its cationic properties, which resembles host defense peptide activity, as described below (Marquis et al., 1982; Ibrahim et al., 1994, 1997; Nishiyama et al., 2001). Fungistatic effects have also been attributed to lysozyme, even at low concentrations (approximately 1 μg/ml for *H. capsulatum*) (Newman et al., 2000). These include: impairment of yeast budding by *C. albicans* and *P. brasiliensis* (Nishiyama et al., 2001; Lopera et al., 2008), decreased production of virulence factors such as aspartyl proteinases (SAP) by *C. albicans* (Wu et al., 1999) and hyphal disruption of *A. fumigatus* (Diamond et al., 1978).

Antileukoprotease (ALP) is a major serine protease inhibitor secreted by Clara and goblet cells at the bronchial epithelium, and by serous cells and submucosal glands in the bronchi (De Water et al., 1986). This 12 kDa cationic non-glycosylated protein exhibits antifungal activity *in vitro* against *C. albicans* and *A. fumigatus* at concentrations comparable to that of lysozyme (Tomee et al., 1997). It remains unclear whether the antifungal mechanism of action is similar to its antibacterial mechanism, which resides mainly in the N-terminal region of ALP (Miller et al., 1989). Its proteinase inhibitory activity, however, is associated with the C-terminal region, which in itself does not have antibacterial activity (Hiemstra et al., 1996; Tomee et al., 1997). Recently, a study by Curvelo et al. (2014) has shown that ALP can also affect *C. albicans* cell membrane stability by inducing several structural changes, most notably irregularities along the cytoplasmic membrane. This finding points toward a mechanism similar to the one displayed by other cationic peptides, such as defensins, known for their ability to permeabilize membranes (see below). *In vitro*, inhibition of *C. albicans* and *A. fumigatus* proteases by ALP decreases fungal adhesion to MA104 epithelial cells from monkey kidneys (Tomee et al., 1997; Curvelo et al., 2014).

Host defense peptides (HDPs) are characterized by their high cationic charge, small size (5–50 amino acids) and amphipathicity. There are two major groups of cationic HDPs secreted into the lung lining: defensins and cathelicidins. Defensins are characterized by three conserved disulfide linkages, which induce a characteristic fold containing a high percentage of β-sheets. A structural subdivision is made between α- and β-defensins based on the location of the cysteine linkages. Four human β-defensins (hBD-1, -2, -3, and -4) are produced by the lung epithelium, either constitutively (hBD-1) or induced upon infection (hBD-2, -3, and -4) (Yanagi et al., 2005; Doss et al., 2010).

The mechanism of action of hBDs has been studied in bacteria, where interaction with cell membranes seems to be an important requirement for their antibacterial activity. However, other effects such as inhibition of DNA, RNA, and protein biosynthesis are also observed and might significantly contribute to growth inhibition and killing (Sahl et al., 2005). hBD-1, hBD-2, and hBD-3 are also known to have antifungal activity but these mechanisms are not well understood (Vylkova et al., 2007). For hBD-1 and hBD-2, the activity against *C. albicans* is dependent on the energy status of the fungal cells, reflecting a requirement for energy-dependent uptake of peptide, but for hBD-3 energy-independent mechanisms are also observed (Vylkova et al., 2006; Krishnakumari et al., 2009). Similar to what is seen for bacteria, all three defensins permeabilize the fungal cell membrane, indicating that membrane destabilization plays an important role in fungal killing (Krishnakumari et al., 2009). Histatin 5, a host defense peptide present in the oral cavity but not in the lungs, binds to specific receptors on the fungal cell membrane, ultimately leading to cell permeabilization. In line with this, Vylkova et al. (2006) have shown that hDB-2 and hBD-3 require Ssa1/-2 surface proteins to kill *C. albicans*, and it seems likely that other peptides or proteins use membrane bound receptors as well.

The mechanism of action of defensins against fungi other than *C. albicans* has been less studied. Contact with metabolically active spores of *A. fumigatus*, for example, increases transcription and secretion of hBD-2 and the recently described hBD-9 by human bronchial epithelial cells. This indicates that several antimicrobial peptides might be released into the lung lining upon fungal infection (Alekseeva et al., 2009; Evans et al., 2010). Levels of α-defensins 1-3 in whole blood seem to increase after exposure to *Candida* spp. yeast cells (Gácser et al., 2014). However, to our current knowledge, no mechanistic data of α-defensins against fungi are available.

Cathelicidins constitute a structurally diverse family of host defense peptides characterized by sharing a similar (cathelin-like) prosequence. In the lung, the only human cathelicidin, hCAP18 is processed by proteases such as elastase, cathepsin G, and proteinase 3 (Sørensen et al., 2001). The processed product is the active peptide LL-37. This peptide is secreted by alveolar macrophages, neutrophils, bronchial glands and by the epithelium of the lung lining (Bals et al., 1998). In a healthy lung, LL-37 can be found at the lung lining at measurable concentrations (Agerberth et al., 1999). *In vitro* antifungal activity of LL-37 has been described for *C. albicans* (den Hertog et al., 2005; López-García et al., 2005; Krishnakumari et al., 2009; Ordonez et al., 2014). Its activity results mainly from permeabilization of the cytoplasmic membrane, although effects on internal organelle membranes have also been described (Ordonez et al., 2014). The only instance in the literature where the role of LL-37 in fungal infections was mentioned is in a study of patients with chronic rhinosinusitis (Lee et al., 2010). In these patients LL-37 levels increased after contact with *A. fumigatus*.

In addition to the described molecules, several other components present in the lung have antifungal activity. These include mainly peptides present in neutrophils that are released upon degranulation, including human α-defensins, histones, Cathepsin G, bactericidal/permeability-increasing protein (BPI), and azurocidin (Newman et al., 2000; Cederlund et al., 2010). No detailed mechanistic antifungal studies have been performed on these molecules but they possess activity against fungi *in vitro*, demonstrating their potential involvement in lung defense against fungal pathogens. An overview of antifungal proteins and peptides is provided in **Table 1**.

**Table 1:** Antifungal activity of soluble innate effector molecules.

Effector molecule	Fungal strains	Proposed mechanism of action
Lysozyme	*H. capsulatum*	Cleavage of chitodextrins
	C. albicans	Cell wall destabilization/permeabilization
	P. brasiliensis	Impairment yeast budding
	*A. fumigatus*	Decrease virulence factors
		Disruption of hyphae
ALP	*C. albicans*	Cell wall destabilization/permeabilization
	*A. fumigatus*	
LL-37	*C. albicans*	Cell wall destabilization/permeabilization
hBD-1	*C. albicans, C. krusei*,	Cell wall destabilization/permeabilization
	*C. parapsilosis, and C. glabrata*	
hBD-2	*C. albicans, C. krusei*,	Cell wall destabilization/permeabilization
	*C. parapsilosis, and C. glabrata*	
	*C. albicans, C. krusei*,	Cell wall destabilization/permeabilization
	*C. parapsilosis, and C. glabrata*	
hBD-4	*C. albicans*	Cell wall destabilization/permeabilization
HNP1	*C. albicans*	Intracellular uptake
	*H. capsulatum*	
HNP2	*C. albicans*	–
	*H. capsulatum*	
HNP3	*C. albicans*	–
	*H. capsulatum*	
Azurocidin	*C. albicans*	–
Cathepsin G	*H. capsulatum*	–
BPI	*H. capsulatum*	–
Histones H1-H4	*C. albicans*	–

#### Opsonization

##### Fungal recognition by SP-A and SP-D

Surfactant proteins A and D (SP-A and SP-D) are two multimeric C-type lectins. In several publications, the structure and processing of SP-A and SP-D have been described in detail (Crouch, 1998; Haagsman and Diemel, 2001). Briefly, these proteins consist of a C-terminal Ca^2+^-dependent carbohydrate recognition domain (CRD), a neck region and an N-terminal collagen-like domain. These glycoproteins are mainly secreted as large octadecameric (SP-A), dodecameric (SP-D), or even higher order oligomeric structures and to a lesser extent as trimeric subunit structures. The low affinity interaction for carbohydrates of the CRDs requires cooperative binding of these domains. Thus, the assembly of collectins to oligomeric structures is essential for increasing the avidity of binding to glycan arrays on the surface of microorganisms. Production and secretion of SP-A and SP-D is mainly attributed to alveolar type II cells and bronchiolar Clara cells, but mRNA expression of both lung collectins is also observed in trachea (Madsen et al., 2000, 2003; van Rozendaal et al., 2001). SP-A strongly associates with the phospholipids present in ‘pulmonary surfactant,’ the protein/lipid mixture produced and secreted by epithelial type II cells, whereas SP-D is not (Persson et al., 1989; Creuwels et al., 1997). This difference affects their distribution: while SP-A will remain largely bound to surfactant lipids at the alveolar lumen and bronchioli, SP-D is relatively more abundant at the upper conductive airways.

**FIGURE 1:**
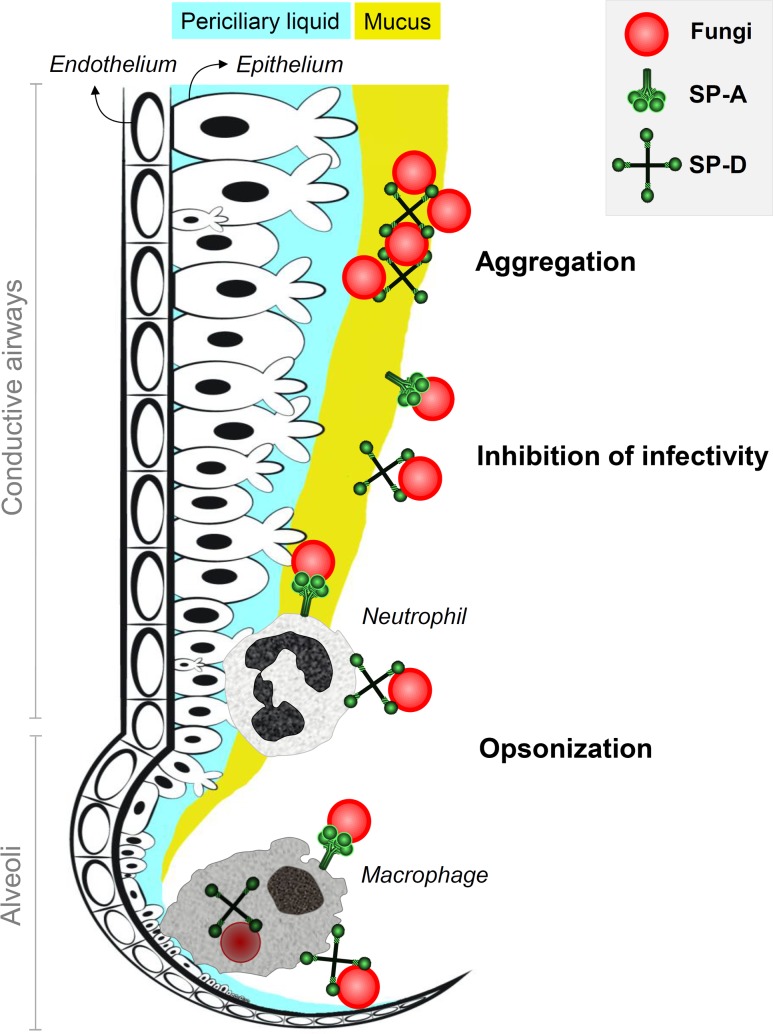
Antifungal roles of SP-A and SP-D at the lung lining. Inhaled fungi can be bound by constitutively expressed lung collectins SP-A and SP-D in the conductive airways and in the alveoli. This generates three mechanisms of protection that contribute to inhibition of fungal infection. Aggregation of fungal particles is mainly facilitated by interactions with SP-D and results in enhanced clearance through mucociliary movement. Binding of collectins to fungal spores also helps to prevent attachment of fungi to pulmonary epithelial cells and thereby inhibits fungal infection. In addition, SP-A and SP-D act as opsonins and mark fungi for recognition, enhanced uptake and improved destruction by neutrophils in the airways and macrophages in the alveoli.

SP-A and SP-D bind to fungi through their CRD in a Ca^2+^-dependent manner. Fungal ligands that are recognized by collectins can be found in **Table 2**. For a more detailed description on fungal interactions with collectins, we refer to an excellent review by Brummer and Stevens (2010). SP-A and SP-D have a preference for binding glycans with a terminal mannose residue but are otherwise quite unspecific (Lu et al., 1992; Haurum et al., 1993). Therefore, it does not rely on the availability of only a single type of polysaccharide on the fungal membrane, explaining the broad fungal binding spectrum of these collectins. Binding to fungal surfaces by oligomeric structures of SP-A and SP-D results in different mechanisms of protection against fungal infection (**Figure 1**). SP-D-mediated aggregation of fungi can facilitate fungal removal, either by mucociliary clearance or by phagocytosis, and helps to prevent infection by blocking fungal attachment to the epithelium (Madan et al., 1997; Yang et al., 2000). Fungal cells coated with either SP-A or SP-D interact differently with immune cells. In some cases, binding of these proteins to fungal cells has shown to enhance macrophage phagocytosis through opsonization and modulate their cytokine secretion (Rosseau et al., 1999). This will be discussed further in the following section.

**Table 2:** Interaction of fungal ligands with SP-A and SP-D.

Fungi	Ligand for SP-A	Effect	Ligand for SP-D	Effect
*C. albicans*	• Sugar moieties at cell wall (van Rozendaal et al., 2001; Geunes-Boyer et al., 2009)	• Reduction of phagocytosis by alveolar macrophages	• Moderate increase in phagocytosis in monocytes and neutrophils	• Downregulation of cytokines in alveolar macrophages (Brummer and Stevens, 2010)	• Mannose Maltose (Rosseau et al., 1997; van Rozendaal et al., 2000; Geunes-Boyer et al., 2009)	• Inhibition of phagocytosis by alveolar macrophages (van Rozendaal et al., 2000; Dennehy and Brown, 2007)
*P. carinii*	• Glycosylation sugars of GPA (Vuk-Pavlovic et al., 2001)	• Enhanced attachment to rat macrophages	• Increased clearance of *P. carinii* infection (Vuk-Pavlovic et al., 2001)	• Glycosylation sugars of Gp-A (O’Riordan et al., 1995)	• Cell wall β-glucans (Vuk-Pavlovic et al., 2001)	• Fungal aggregation
• Increased binding to macrophage surface	• Decreased fungal internalization (Atochina et al., 2004)
*H. capsulatum*	• Sugars at the cell surface (not identified) (Dennehy and Brown, 2007)	• Fungal permeabilization	• Sugars at the cell surface (not identified)	• Fungal permeabilization
*A. fumigatus*	• Mannose Maltose	• Enhanced phagocytosis and killing by macrophages and neutrophils (Philippe et al., 2003)	• Mannose Maltose	• Enhanced phagocytosis and killing by macrophages and neutrophils (Philippe et al., 2003)
*C. neoformans*	?	?	• Glucuronoxylomannan (GXM)	• Aggregation of acapsular *C. neoformans* (van de Wetering et al., 2004; Geunes-Boyer et al., 2009)
			• Sugars at the cell surface	

##### Effects of SP-A and SP-D opsonization on the antifungal activity of phagocytic cells

Collectins are known to interact with phagocyte receptors as well as with fungal ligands and can thereby be of influence during an encounter between them. Brown (2011) describes in detail how fungal pathogens are recognized by phagocytes through several pattern recognition receptors (PRR), which comprise Toll-like receptors (TLRs) as well as C-type lectin receptors (CLRs). The structures present in the fungal cell wall that can be recognized by PRRs include mannan, phospholipomannan, *O*-linked mannan, glucuronoxylomannan, galactomannan, and β-glucans (Netea et al., 2006; Willment and Brown, 2008; Figueiredo et al., 2011; Bourgeois and Kuchler, 2012). Some PRRs are known to interact with SP-A and SP-D directly such as TLR2, TLR3 and TLR4, gp-340, CD91/calreticulin, and SIRPα (Holmskov et al., 1999; Gardai et al., 2003). As for CLRs expressed at the surface of phagocytes, one can imagine that these compete with SP-A and SP-D for similar binding sites at the fungal surface.

Recognition by PRRs initiates phagocytic uptake. Internalized fungi are directed to acidic compartments, called lysosomes, which further fuse to form phagolysosomes. Here, a cocktail of hydrolytic enzymes, host defense peptides, reactive oxygen species, and reactive nitric oxide species are in charge to destroy the internalized fungi (Ibrahim-Granet et al., 2003; Philippe et al., 2003; Netea et al., 2008). Collectin-opsonization of fungal cells usually enhances phagocytosis. However, enhanced phagocytosis could either be favorable or unfavorable for fungal clearance depending on the fungus involved. For example, increased phagocytosis by macrophages and neutrophils has shown to improve killing of *A. fumigatus* (Madan et al., 1997), while increased phagocytosis can be favorable for survival of *C. neoformans* and *H. capsulatum*, likely indicating how certain fungi have evolved to withstand (certain aspects of) the immune response within the lung. Extracellular growth of *C. neoformans* can be inhibited by macrophages while internalized *C. neoformans* is able to survive (Flesch et al., 1989). For *H. capsulatum*, internalization by macrophages allows fungal survival while internalization by neutrophils and dendritic cells has proven to be fungistatic and fungicidal, respectively (Deepe et al., 2008).

Interestingly, for *Pneumocystis carinii*, collectin binding actually decreases phagocytosis. SP-D binding to the 120 kDa mannose-rich glycoprotein (GPA) of *P. carinii* blocks the fungal cell from interacting with macrophage mannose receptors that are responsible for phagocytosis (O’Riordan et al., 1995; Vuk-Pavlovic et al., 2001). Decreased phagocytosis in the presence of SP-D was also observed for *C. albicans* (van Rozendaal et al., 2000). SP-A also binds to GPA of *P. carinii* but actually enhances its association to alveolar macrophages (Atochina et al., 2004). These opposing effects of SP-A and SP-D, observed for *P. carinii*, are hard to explain with the current knowledge and one can only speculate about these outcomes. Two reasons have been proposed: one is that SP-A interaction with macrophage receptors may increase phagocytosis, counteracting GPA blockage; another hypothesis suggested by Yong et al. (2003) is that the tertiary structures of SP-A and SP-D differ so strongly that they may promote different outcomes even though binding the same ligand.

#### Immunomodulation

Until now immunomodulatory effects of host defense molecules have been strongly underestimated. In the fight against bacterial infections, immune modulation by host defense molecules plays an important role and it is likely that this also accounts for fungal clearance. A broad range of immunomodulatory functions has been described for several of the innate defense molecules in the airway surface liquid (ASL).

**FIGURE 2:**
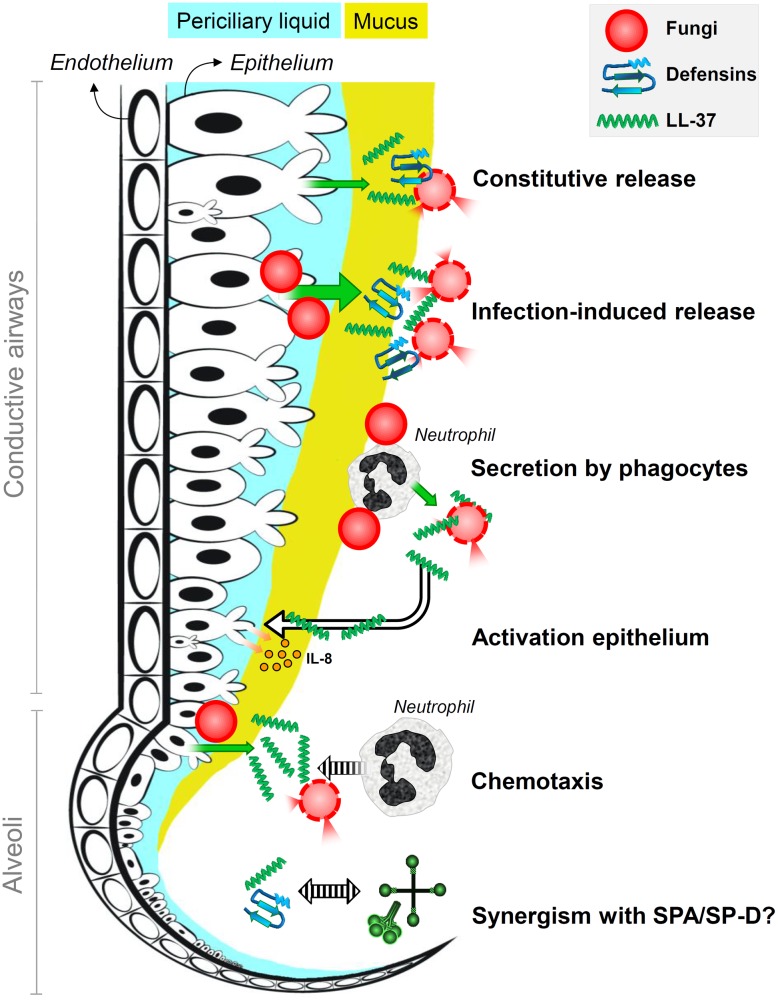
Antifungal roles of host defense peptides at the lung lining. It is the schematic representation of the antifungal roles of defensins and the human cathelicidin LL-37 in human lungs. Fungi are killed by membrane destabilization and permeabilization due to interactions with defensins and LL-37 that are constitutively released into the lung lining fluid by airway epithelial cells; epithelial secretion of these peptides is induced upon infection. Phagocytic cells that encounter fungal spores, in particular neutrophils, secrete LL-37 that results in enhanced killing and epithelial expression of interleukin-8. Infection-induced secretion of LL-37 also contributes to attracting neutrophils that help to clear the infection. Host defense peptides may also act synergistically with surfactant proteins SP-A and SP-D.

Host defense molecules could prepare immune cells for encounters with fungi in the healthy lung. A study by Scott et al. (2002) shows that LL-37 may contribute to the immune response against bacteria by limiting host cell damage and increasing phagocyte recruitment to infection sites. During infection, an increase in secretion of several defense molecules is observed. Human lactoferrin, for example, triggers the transcription of host defense peptides by bovine tracheal epithelial cells (Velliyagounder et al., 2003). Host defense molecules can increase protection in areas near the infection and additionally attract phagocytic cells. For instance, LL-37 secreted by epithelial cells is known to act as a chemoattractant for neutrophil recruitment (De et al., 2000). LL-37 produced by PMNs has also been suggested to activate the epithelium resulting in IL-8 release (Tjabringa et al., 2003). An overview of the activities of HDPs is given in **Figure 2**.

Collectins can induce macrophage actin changes (Veillette et al., 1998; Gardai et al., 2003), integrin induction (Senft et al., 2007), and increase cellular receptors on phagocytes (Kuroki et al., 2007). These collectin-induced modifications might be essential for proper phagocyte activation. In fact, macrophages alone are not able to clear *A. fumigatus* in an *in vitro* cell-based infection model. *In vitro*, SP-A downregulates the production of pro-inflammatory cytokines by alveolar macrophages evoked by *C. albicans* (Rosseau et al., 1999). Additionally, SP-A interacts with macrophages affecting their response to LPS, and decreases macrophage TNFα production by interaction with TLR2 (Fisher et al., 2002; Murakami et al., 2002).

Cell receptors and signaling pathways activated by collectins have been studied mainly in macrophages, but they are also known to trigger lymphocyte proliferation and leukocyte recruitment (Madan et al., 1997; Crouch, 1998; Fisher et al., 2002). Several cell receptors have been found to be responsible for SP-A- and SP-D-induced signaling in inflammation. Gardai et al. (2003) showed that the CRD domain of both collectins bind to the inhibitory signal regulatory protein α (SIRPα) at the macrophage surface, while the collagenous domain binds to calreticulin/CD91. Binding to SIRPα blocks pro-inflammatory responses and decreases phagocytosis of apoptotic cells, while binding to calreticulin/CD91 increases pro-inflammatory responses, chemotaxis, and macrophage phagocytosis. Additionally, another pathway for the immunomodulatory function of SP-D has been recently described by Olde Nordkamp et al. (2014), where the collagenous domain of SP-D was found to bind the Leukocyte-Associated Ig-like Receptor 1 (LAIR-1) at the neutrophil surface, inhibiting the production of FcαR-mediated reactive oxygen. These examples show the complexity of the immunomodulatory role of these soluble lung effector molecules, with sometimes seemingly opposing (pro- and anti-inflammatory) effects. It is even likely that the described examples only constitute a fraction of all immunomodulatory routes, which emphasizes again that a better understanding of the interaction between all components is required to predict the outcome of an immune response.

### Effects of Lung Environment on Antifungal Activities

Most of the antifungal components and mechanisms described above are derived from *in vitro* studies and, in most cases, have been determined in a one-to-one set-up. For example, the antimicrobial activity of host defense peptides such as LL-37 or defensins is determined by incubating fungi with the peptide in a specified buffered solution after which the viability of the fungi is monitored in time. Although valuable observations can be made this way, one should be careful in extrapolating these activities to an *in vivo* situation. The complex composition of the ASL will likely affect the activity of individual innate immune effector molecules. Several factors can modulate the activity of soluble effector molecules: (1) pH, (2) ionic strength, (3) divalent cations, and (4) interactions with mucus.

#### pH

In general, the ASL of a healthy lung is known to have a neutral pH of 6.94 ± 0.03 (Johansson et al., 1998; Nakayama et al., 2002; Olde Nordkamp et al., 2014) and a decrease in pH affects the ability of the ASL to eliminate pathogens (Pezzulo et al., 2012), likely due to a reduced activity of several defense molecules (Berkebile and McCray, 2014). This can happen during infection at specific locations inside the lung, or during certain diseases such as cystic fibrosis. From what is described for bacteria, a low pH may reduce the activity of LL-37 by perturbing its α-helical conformation and interaction with membranes (Johansson et al., 1998). Low pH also decreases HBD-1 secretion by epithelial cells (Nakayama et al., 2002), phagocyte chemotaxis, and ROS production. However, specific effects of pH with respect to their antifungal activities have not been described yet (Lardner, 2001; Ng et al., 2004). On the other hand, the Ca^2+^-binding properties of SP-D and SP-A are mostly retained at pH values as low as 5.0 (Haagsman et al., 1987; Crouch, 1998). It is not known, however, whether pH changes may affect phagocyte interactions with these collectins (Rosseau et al., 1999). Whether such a low pH could exist locally in a healthy lung is debatable, but phagocytic responses might generate acidic microenvironments that could reach low pH values.

#### Ionic Strength

The activity of most HDPs is based on their high cationic charge and the ASL ionic concentration can therefore strongly affect their activities. The major component contributing to the ionic strength of the ASL is NaCl, present in a concentration of approximately 100 mM at the ASL of the healthy lung (Jayaraman et al., 2001). *In vitro*, such salt concentrations significantly diminish the direct antifungal activity of several host defense peptides including defensins and LL-37 (Vylkova et al., 2007; Krishnakumari et al., 2009; Coorens et al., 2017), lysozyme and ALP (Tomee et al., 1997), although the antifungal activity of HBD-3 appears less sensitive as compared to that of other defensins (Turner et al., 1998; Krishnakumari et al., 2009). The high amount of antimicrobials needed to exert adequate antifungal killing at physiological ionic concentrations does not correspond with the concentrations normally found at the ASL. However, there is evidence that at lower than minimum inhibitory concentrations, innate effector molecules can still affect fungal growth. Lysozyme, for example, can still decrease *C. albicans* growth, morphology transition, and protein production at inflammatory concentrations around 400–800 μg/ml (Lopera et al., 2008). Additionally, lysozyme inhibits *C. albicans* virulence-related proteins at sub-inhibitory concentrations (10 μg/ml) (Wu et al., 1999).

The collectins SP-A and SP-D are also affected by ionic concentrations but in opposite ways. While purified SP-A precipitates at physiological salt concentrations, SP-D requires physiological salt concentrations to maintain its structure and functionality. This could correlate with their localization *in vivo*. Inside the lung, SP-A is mainly forming a complex with surfactant lipids whereas most of SP-D locates in the aqueous salt-containing sub-phase (Crouch, 1998). The localization of these two collectins could make the difference for their availability to bind fungal cells and how they modulate presentation to phagocytes. SP-D has shown to be able to bind fungal spores in BALF (Yang et al., 2000). Nevertheless, a fraction of SP-A produced by Clara cells and submucosal glands at the conductive airway may not be bound to lipids (as is the case for SP-A at the alveoli) and therefore could bind to inhaled microorganisms (Khoor et al., 1993). It is unclear though, to what extend SP-A might affect macrophage activation.

#### Divalent Cations

The presence of divalent cations at the lung lining, such as Mg^2+^, Fe^2+^, and Ca^2+^, strongly influences ASL antifungal activity. *In vitro* concentrations of ∼5 μM Ca^2+^ or ∼25 μM Mg^2+^ are sufficient to abrogate the candidacidal activity of defensins, especially HBD-1 and HBD-2 (Krishnakumari et al., 2009). The antifungal activity of LL-37 is also decreased by Ca^2+^ at concentrations as low as 1 μM (Ordonez et al., unpublished data). This is similar to what has been observed for its antibacterial activity where the minimum inhibitory concentration of LL-37 for *Escherichia coli* increased 10-fold in the presence of 1 mM CaCl_2_ (Turner et al., 1998). There are also several chelators present at the ASL that sequester divalent cations as a defense against pathogenic organisms. Lactoferrin, for example, is a monomeric multifunctional glycoprotein of 80 kDa secreted by lung epithelium and submucosal glands into the ASL. There, lactoferrin is found in high concentrations (Dubin et al., 2004; Viejo-Díaz et al., 2004). This protein is best known for its ability to trap iron ions. According to Ding et al. (2014), iron ions are essential for fungal virulence. In line with these observations, Soukka et al. (1992) described candidacidal effects of lactoferrin that were dependent on iron ion sequestering since Fe^3+^-saturated lactoferrin completely lost its ability to kill *C. albicans*. Transferrin, a siderophore present in the deep lung, is also known to limit iron availability, thus inhibiting microbial growth. Although this siderophore will chelate ions, several pathogenic bacteria such as *P. aeruginosa* are known for their ability to use them as a source of ions (Cornelis and Dingemans, 2013). Interestingly *C. neoformans* is also able to use transferrin as a source of iron (Saikia et al., 2014).

#### Mucus

Mucus is a complex structure mainly composed of highly glycosylated proteins called mucins. This structure covers the epithelial surface throughout the conductive airways, thereby serving as a barrier against inhaled microorganisms. The regulation of mucus secretion by epithelial cells is tightly linked to pH and ionic concentrations at the ASL. To date, 20 genes coding for mucosal proteins have been described (Rose and Voynow, 2006). A total of 14 of these mucins are expressed in the lung as demonstrated by RNA expression, with MUC5AC and MUC5B being the most abundant components of the respiratory mucus (Hovenberg et al., 1996). Interaction of mucus with cationic peptides is likely to occur since mucins are negatively charged. It is known that LL-37 and HBD-2 bind mucins in the gastrointestinal tract (Felgentreff et al., 2006; Cobo et al., 2015). Mucins are highly glycosylated, and this enables their interaction with lectins, such as SP-D. Although SP-D is produced mainly in the deep lung, its localization in the aqueous phase might enable it to travel throughout the conductive tract. Nevertheless, low expression of this protein was also observed at the level of the trachea (Madsen et al., 2000; Herias et al., 2007). Whether SP-D interactions with mucus at the conductive airway interfere with SP-D binding to pathogens remains to be studied.

### Combined Immune Defenses against Fungal Pathogens

Previously, we have discussed how ASL conditions may affect the interactions between antimicrobial molecules and fungal pathogens. It is easy to imagine that the concentrations needed for exerting antifungal activity would have to be higher than the ones described in *in vitro* experiments. It can be speculated though that in the healthy lung, the concerted action of several antimicrobial molecules may help to protect against fungal colonization. In this scenario, we have to consider synergistic, additive, and antagonistic activities of the different soluble effector molecules present.

*In vitro*, combined effects have already been described against bacteria, but these are less understood for fungi. For bacteria, combinations of lactoferrin, lysozyme, ALP, and LL-37 appear to be synergistic, while combinations with defensins seem to have only an additive effect (Bals et al., 1998; Singh et al., 2000). However, it remains to be investigated to what extent these effects occur at the ASL with salt concentrations of approximately 100 mM, since Singh et al. (2000) show loss of synergism at salt concentrations as low as 45 mM. Even though combinatorial fungicidal activities between antimicrobials have not yet been described, there is some evidence for a synergistic effect with antifungal drugs (Wakabayashi et al., 1998; Kuipers et al., 1999). Lactoferrin acts synergistically with amphotericin B, 5-fluorocytosine, and more strongly with fluconazole against *C. albicans* (Wakabayashi et al., 1998). This synergism seems to be related to the different mechanisms of action between fluconazole and lactoferrin. While fluconazole inhibits ergosterol synthesis, lactoferrin directly interacts with the membrane, making it unstable. The result is a 50% reduction in the concentration of fluconazole needed for complete growth inhibition in the presence of lactoferrin.

Interactions of surfactant proteins SP-A or SP-D with other antimicrobial components of the ASL have been widely overlooked. It seems plausible that these collectins can interact with defensins and LL-37 due to the cationic nature of these peptides (Doss et al., 2010). The results of these interactions could be either positive or negative for fungal clearance. On one hand, assuming that interactions are weak enough, SP-A/SP-D-bound peptides can be released and bind to fungal membrane targets. On the other hand, if binding between these molecules is strong enough, either peptide activity or sugar recognition by the surfactant protein could be reduced. In this respect, it is worth to mention that while the antiviral activity of SP-D is not affected by binding to β-defensins 5 and 6, binding with neutrophil defensins 1 and 2 actually leads to decreased antiviral activity of SP-D (Doss et al., 2009, 2010). This is most likely due to stronger interactions between neutrophil defensins and SP-D that interfere with recognition by SP-D of viral target proteins.

## Conclusion

Altogether it is clear that physiological conditions at the lung lining may strongly contribute to the antifungal activity of soluble effector molecules. Most of the direct antifungal activities of host defense peptides are dependent on factors such as pH and divalent cation concentrations at the lung lining. It is important to realize that interactions among different innate immune molecules, and the way they interact with host cells, are of major importance in controlling fungal intruders. Therefore, it is essential that the antifungal activity of innate immune molecules should be tested in more complex environments resembling their natural environment *in vivo*.

Several experimental models that mimic the lung surface more closely are currently being developed and optimized. The availability of several alveolar and bronchiolar epithelial cell lines is aiding in this process (Foster et al., 1998; Grainger et al., 2009; Escobar et al., 2016). Usage of a cell-based system that includes several relevant immune components like salts, mucus, and immune cells has become within reach. Such a system will provide a good *in vitro* model to test antifungals in a more realistic environment. Hope et al. (2007) described a model of the lung consisting of a bilayer of epithelial and endothelial cells. Surprisingly, this study showed that neither the tested antifungal compound, nor the use of macrophages could eliminate an *A. fumigatus* infection but rather the combination of both: an effect not observed in simpler models.

In addition, the emergence of new technologies such as the lung-on-a-chip system could be of great help to dissect the antifungal mechanisms and importance of the innate immune system *in vitro* under circumstances that mimic the human lung (Esch et al., 2015). This microdevice makes use of endothelial and epithelial cells grown on a polymer support that produces a polarized cell layer with one side submerged in liquid (blood flow) and the other side exposed to air. This setting could help mimic the conditions during a fungal infection, especially at later stages where hyphal growth is believed to reach the endothelium. Finally, a recent report described the development of human lung organoids, resembling the upper airways (Dye et al., 2015). The use of these sophisticated model systems will ultimately lead to a more thorough understanding of the innate defense against fungi, and may contribute to the development of novel, more effective drugs for the treatment for fungal infections in humans.

## Author Contributions

SO performed the conception and design of the review and the revision and analysis of the literature, and drafted figures. EV performed the conception and design of the review and the revision and analysis of the literature. ME revised the manuscript and drafted figures. HH performed design of the review, revised the manuscript, and gave final approval.

## Conflict of Interest Statement

The authors declare that the research was conducted in the absence of any commercial or financial relationships that could be construed as a potential conflict of interest.
